# A practical approach to goal-directed echocardiography in the critical care setting

**DOI:** 10.1186/s13054-014-0681-z

**Published:** 2014-12-01

**Authors:** Patricia E Walley, Keith R Walley, Ben Goodgame, Vivek Punjabi, Demetrios Sirounis

**Affiliations:** Cardiac Echo Laboratory, St Paul’s Hospital, University of British Columbia, Vancouver, V6Z 1Y6 Canada; Intensive Care Unit, St Paul’s Hospital, University of British Columbia, Vancouver, V6Z 1Y6 Canada; Centre for Heart Lung Innovation, St Paul’s Hospital, University of British Columbia, Vancouver, V6Z 1Y6 Canada; Cooper University Hospital, Camden, NJ 08103 USA

## Abstract

Urgent cardiac ultrasound examination in the critical care setting is clinically useful. Application of goal-directed echocardiography in this setting is quite distinct from typical exploratory diagnostic comprehensive echocardiography, because the urgent critical care setting mandates a goal-directed approach. Goal-directed echocardiography most frequently aims to rapidly identify and differentiate the cause(s) of hemodynamic instability and/or the cause(s) of acute respiratory failure. Accordingly, this paper highlights 1) indications, 2) an easily memorized differential diagnostic framework for goal-directed echocardiography, 3) clinical questions that must be asked and answered, 4) practical issues to allow optimal image capture, 5) primary echocardiographic views, 6) key issues addressed in each view, and 7) interpretation of findings within the differential diagnostic framework. The most frequent indications for goal-directed echocardiography include 1) the spectrum of hemodynamic instability, shock, and pulseless electrical activity arrest and 2) acute respiratory failure. The differential diagnostic categories for hemodynamic instability can be remembered using the mnemonic ‘SHOCK’ (for Septic, Hypovolemic, Obstructive, Cardiogenic, and (K) combinations/other kinds of shock). RESP-F (for exacerbation of chronic Respiratory disease, pulmonary Embolism, ST changes associated with cardiac or pericardial disease, Pneumonia, and heart Failure) can be used for acute respiratory failure. The goals of goal-directed echocardiography in the unstable patient are: assessing global ventricular systolic function, identifying marked right ventricular and left ventricular enlargement, assessing intravascular volume, and the presence of a pericardial effusion. In an urgent or emergent setting, it is recommended to go directly to the best view, which is frequently the subcostal or apical view. The five views are the subcostal four-chamber view, subcostal inferior vena cava view, parasternal long axis view, parasternal short axis view, and the apical four chamber view. Always interpret goal-directed echocardiographic findings in the context of clinically available hemodynamic information. When goal-directed echocardiography is insufficient or when additional abnormalities are appreciated, order a comprehensive echocardiogram. Goal-directed echocardiography and comprehensive echocardiography are not to be used in conflict with each other.

## Introduction

Important guidelines have recently been published for the use of echocardiography in the critical care setting [1–6] and a large number of publications have investigated important aspects of echocardiography in critically ill patients, in particular the goal-directed approach [1–6]. Here we review the current use of, and a problem-oriented practical guide to, goal-directed echocardiography in the critical care setting. Effective management of critically ill patients requires rapid assessment and clear goals. Even the expert practitioner, with knowledge and skills that allow an extensive and comprehensive examination, must focus on the urgent problem at hand and may benefit from a readily remembered diagnostic framework and a goal-directed approach [7,8]. Compared with a conventional comprehensive echocardiogram [9], goal-directed echocardiography has a more limited scope [10] that specifically addresses the potential causes of 1) the spectrum of hemodynamic instability, shock, and pulseless electrical activity (PEA) arrest and 2) acute respiratory failure. Portability, the time needed to acquire data, ease of use, relatively low cost, and the fact that it is used as an adjunct to the physical examination are major benefits of it [11]. Goal-directed echocardiography can be used instantly for goal-directed therapy in the setting of a hemodynamically unstable patient [12]. Another major benefit of goal-directed echocardiography is that it can be used serially [13] to assess response to interventions in a ‘real-time’ manner and thus guide ongoing therapies [8,14]. Importantly, we emphasize that goal-directed echocardiography should always be interpreted in view of clinically available hemodynamic information (arterial pressures, central venous pressure, use of vasopressor/inotropic drugs, ventilator settings, urine output, and so on) and respiratory data (chest radiography, arterial and venous oxygen saturation, shunt fraction, dead space, acid–base status, and so on).

## Definition and scope of goal-directed echocardiography

Goal-directed echocardiography is different from the comprehensive echocardiograms performed by fully trained, experienced, and certified cardiac sonographers, cardiologist echocardiographers, and advanced critical care echocardiographers [3–5,9]. Comprehensive echocardiograms are beyond the scope of beginners, who will use only a few echocardiographic views. A key challenge for intensivists is to know their (narrow) field of competence while using transthoracic echocardiography in the ICU setting. Advanced practitioners are capable of measuring many more parameters designed to assess many additional aspects of cardiac structure and function and requiring more time, additional views, additional tools (intravenous contrast, maneuvers), and a higher level of qualifications and training in the acquisition and interpretation of images [3–5,9,15,16].

In conducting goal-directed echocardiography, the most technically difficult patients are imaged in the most challenging situations; hence, diagnostic shortcomings are exaggerated in this population because of, for example, lung interference, mechanical ventilation, positioning limitations, and the urgency to acquire the information for immediate treatment. Current research suggests that training internists and residents with limited previous ultrasound skills is feasible and can result in accurate assessments when using goal-directed echocardiography [11,17–19]. It is important to recognize, however, when the necessary images cannot be acquired or are insufficient for accurate interpretation. It is then necessary to order a comprehensive echocardiogram to further assess potential findings and findings that fall outside the scope of goal-directed echocardiography [1] (for example, valvular abnormalities, wall motion abnormalities, diastolic dysfunction, abnormal anatomy or other abnormal echocardiographic features). An ‘incidental’ finding of a significantly thickened aortic valve should be noted due to possible implications during resuscitation attempts [20]. When these additional observations are appreciated or suspected, a full comprehensive echocardiographic examination is indicated as soon as feasible.

The issues of how goal-directed echocardiography is different from conventional echocardiography and when goal-directed echocardiography alone is sufficient are the subject of careful discussion in the recent publication by Spencer and colleagues [1]. The American Society of Echocardiography Expert Consensus Statement specifically discusses the role of ‘goal-directed echocardiography when echocardiography is not promptly available’. We address a narrow aspect of these guidelines in detail, specifically the use of goal-directed echocardiography to distinguish between differential diagnostic categories contributing to 1) hemodynamic instability, shock, and PEA arrest requiring immediate therapeutic decisions and interventions - the central indication for goal-directed echocardiography in the critical care setting - and 2) evaluation of acute respiratory failure. In every case the operator should consider whether a subsequent comprehensive echocardiographic examination would contribute additional information necessary for ongoing care.

## Guidelines for training

An expert round table led by the European Society of Intensive Care Medicine has recently developed an international consensus statement on guidelines for training and accreditation which have been widely adopted [3]. Earlier published statements [15,21], the Canadian Society of Echocardiography [22], and the American Society of Echocardiography [1] have also addressed aspects of training.

While cardiologists and cardiac sonographers are generally the most highly trained and experienced operators, there are emerging important roles for different kinds of providers. Goal-directed echocardiography is often performed by physicians who are not trained in acquisition and interpretation of comprehensive echocardiograms and who come from diverse backgrounds and different clinical experiences. For these physicians guidelines for goal-directed echocardiography training in the ICU are much more focused and are currently in development [1,15,19,23]. Transesophageal echocardiography also plays a role in the ICU but is not explored in this review as it is appropriately performed by skilled and highly knowledgeable operators conducting sufficient examinations every year to maintain competency [2]. Training guidelines for transesophageal echocardiography in the critical care setting have recently been published [3].

### Developing an efficient training program

Application of ultrasonography by intensivists and other clinicians is increasing and widespread use is inevitable [10,24,25]. Technical training for performing goal-directed echocardiography and interpretation takes time, practice, and supervision [11,26,27]. It is imperative to recognize technical and interpretive limitations. The ‘trainees’ are frequently physicians with very busy schedules who are accustomed to being experts [28]. The recent International Consensus Statement on training standards for advanced critical care echocardiography are clear that a training program should be rigorous and include competence-based testing [3]. Other organizations have endorsed concordant views [1]. For example, the American College of Emergency Physicians suggests that didactic training, extensive hands-on experience, and expert review should be included in every case [29] and partnership with a comprehensive echocardiography laboratory is required [1]. This should be supplemented with a continuing medical education program [2] and there should be a formal certification process to ensure competence in technical skills and interpretation [3,30] as poor training may result in adverse consequences [31].

## Indications

Goal-directed echocardiography has now become a reliable tool and can be performed within minutes [17] and is useful for assessing the spectrum of hemodynamic instability, shock, and PEA arrest [2,32–34]. The differential diagnostic categories for hemodynamic instability can be remembered using the mnemonic ‘SHOCK’ (for Septic, Hypovolemic, Obstructive, Cardiogenic, and (‘K’) combinations or other kinds of shock) . ‘S’ stands for the broad category of septic shock due to infection or similar distributive shock from non-infectious causes. Hypovolemia (H) is identified by changes in inferior vena cava (IVC) diameter and by small, underfilled ventricles. Obstructive shock (O) is most frequently due to pericardial tamponade or pulmonary embolism, which manifest differently (see below). Cardiogenic shock (C) is usually characterized by dilated and/or poorly contractile ventricles or marked valvular dysfunction recognized by using color Doppler imaging [5]. When the clinical picture and goal-directed echocardiography do not fit clearly into one of these major categories, other possibilities must be considered - ‘K’, remembered phonetically, for ‘combinations’ and other ‘kinds’. That is, shock may be due to combinations of the above etiologies and rarely due to other disease processes such as adrenal insufficiency, neurogenic shock, and so on.

Goal-directed echocardiography can be combined with thoracic ultrasound imaging [35] and improves diagnostic accuracy over conventional imaging for patients presenting with acute respiratory failure [36]. The most common causes are captured by the mnemonic RESP-F - exacerbation of chronic Respiratory disease, pulmonary Embolism, ST changes associated with acute cardiac or pericardial disease, Pneumonia, and heart Failure. Lung and thoracic imaging (beyond the scope of this review) are an essential component of this examination to additionally identify pleural effusion, pneumothorax, and consolidated or edematous lung [35,36].

## Specific goals

The goal of goal-directed echocardiography is to identify the cause (mnemonic SHOCK) of hemodynamic instability, shock, or PEA arrest and to expedite directed therapies [1,32–34]. To accomplish this, the specific goals are the systematic assessment of 1) left ventricule (LV) systolic function (cardiogenic) [2,17,37], 2) right ventricule (RV) size and function [38] (hypovolemia, obstruction [39,40], cardiogenic), 3) pericardial effusion, potential signs of hemodynamic compromise (obstruction), and 4) size and distensibilty of the IVC for evaluation of volume status (hypovolemia) [14].

A structured approach to accomplishing the goals of goal-directed echocardiography involves specifically asking and addressing key practical questions, as follows. First, does the LV appear significantly dilated or not? Does the LV function appear ‘significantly’ impaired or not? Second, is the RV dilated or not? Third, is there evidence of hypovolemia (small LV, narrow IVC (<21 mm) and collapsing >50% with spontaneous respiration). In the more severe cases of hypovolemia, and in a non-mechanically ventilated patient, an IVC diameter of ≤1 cm usually indicates preload responsiveness [41]. Fourth, is pericardial effusion present (moderate to large)?

Goal-directed echocardiography should note the presence of additional abnormalities but the diagnostic pursuit of these additional findings is accomplished by a comprehensive echocardiographic examination. Specifically, the presence of significant valvular abnormalities, wall motion abnormalities, LV aneurysm, RV hypertrophy, cardiac masses, thrombus, diastolic dysfunction, or a dilated ascending aorta with potential dissection should be pursued with a comprehensive echocardiographic examination, when recognized [1].

## Equipment set up

### Transducer

Use a low-frequency transducer for adequate penetration to image cardiac structures. Transducers with a higher frequency have better resolution at the expense of deeper tissue penetration. Transducers with a lower frequency have better penetration at the expense of higher resolution. The ‘footprint’ or size of the low-frequency transducer head is designed to be small enough to better fit between the intercostal spaces.

### Initial settings

**Figure 1:**
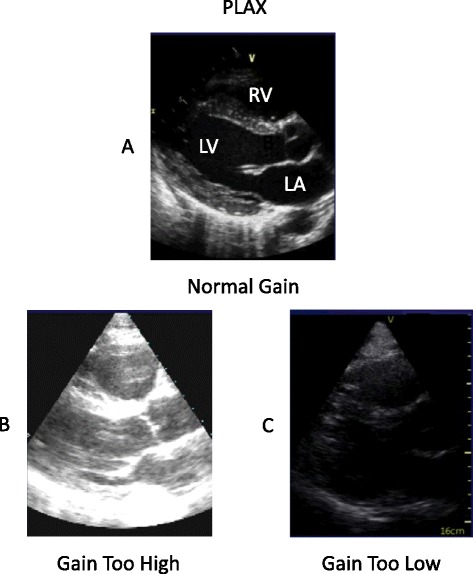
**The parasternal long axis (PLAX) view. (A)** Normal gain. **(B)** Gain too high; with the gain too high, echocardiographic anatomy data may be obliterated. **(C)** Gain too low; with the gain too low, echocardiographic anatomy data may be incomplete. LA, left atrium; LV, left ventricle; RV, right ventricle.

Depth is increased to 20 to 24 cm to identify pericardial effusion and the incidental finding of pleural effusion. Decrease ‘depth’ to 14 to 16 cm for a full screen cardiac image. Continually adjust the ‘focus’ control to the anatomy imaged for better resolution. Gain should be adjusted to see endocardial borders clearly (Figure 1A). Be careful not to ‘over gain’ (too bright, washes out image) or ‘under gain’ (weak echocardiographic images) (Figure 1B,C).

## Views

Goal-directed echocardiography comprises four main views. In an urgent or emergent setting, ‘go for the gold’. Apical and subcostal views are frequently most useful [42]. That is, if unable to obtain an adequate image in one view, move on to the next view, when necessary. In critical care settings, you may not always have the electrocardiogram on the echocardiographic monitor to provide a timing marker. For timing of the cardiac phase, pay attention to whether the mitral/tricuspid valves are open (diastole) or closed (systole). Remember, you must see endocardium to accurately assess ventricular function for all the views listed.

### Subcostal view

**Figure 2:**
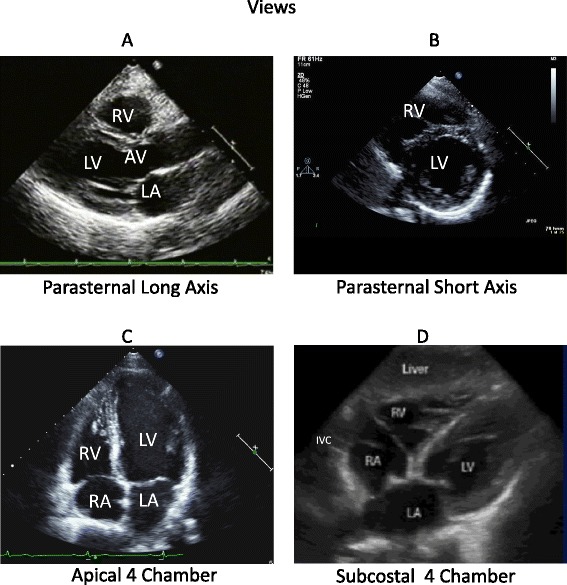
**Standard four views of goal-directed echocardiography. (A)** Parasternal long-axis view. Horizontal view of the heart, including the ascending aorta, the aortic valve (AV), the right ventricle (RV), the left ventricle (LV), the left atrium (LA), frequently excluding the apex, and the pericardium. **(B)** Parasternal short-axis view. A transverse view of the mid-LV at the level of the papillary muscles, including the RV, and the pericardium. **(C)** Apical four-chamber view, including the LV, RV at the RV inlet, trabeculated apical, and the infundibulum or smooth myocardial outflow regions, LA, right atrium (RA), mitral valve, tricuspid valve, and pericardium. **(D)** Subcostal four-chamber view. A more perpendicular orientation of the LV, RV, LA, RA, mitral and tricuspid valves, interatrial septum, pericardium, inferior vena cava (IVC), and liver.

The subcostal view (Table 1 and Figure 2) provides more information than any other individual view and, as a result, addresses more of the key questions. In particular, volemia is best addressed in this view and multiple aspects (IVC collapse [14,43], RV and LV diastolic size) are sometimes evident from this one window. The IVC can be used for estimating central venous pressure, reflective of right atrial pressure [14,43,44]. The ratio of inspiratory and expiratory changes in diameter is very useful in evaluating volume status [45–47]. The subcostal view, as with the apical four-chamber (A-4) view, is frequently the best view to appreciate the presence of a pericardial effusion and associated echocardiographic evidence of hemodynamic compromise [2]. This view is frequently used for a pericardiocentesis. It is sometimes difficult to differentiate pericardial fluid, fat, clot, or thrombus. A small, focal or loculated pericardial effusion may not be visualized nor easily ‘tapped’. When results are in question, order a comprehensive echocardiographic examination.

**Table 1:** **Subcostal and apical four-chamber view**

	**Subcostal view**	**Apical four-chamber view**
Goal	Volume status - IVC collapse. LV and RV size and function. Pericardial effusion. Right pleural effusion	LV size and function, RV size and function, and pericardium. RV/LV ratio. Pericardial effusion. Left pleural effusion. Aortic and mitral valve thickening. Significant valvular regurgitation - color Doppler
Patient position	Supine with pillow under knees to relax abdominal wall	Far left lateral, when possible
Initial transducer placement	Transducer at the subxyphoid position and angled slightly medially, pointing the transducer (soundwave beam) to the patient’s left side. Transducer marker at approximately 3 o’clock. Transducer almost flat against the abdominal wall, approximately at a 15 degree angle	Start at the apex. Rotate the transducer clockwise with the tranducer marker at approximately 3 o’clock and angle the transducer or sound wave beam up through the apex of the heart or right shoulder
Search for the best window	Transducer angled laterally to image the apex of the RV, upper right side of screen, LV on the lower right side of screen, right atrium on the upper left side of screen, and left atrium on the lower right side of screen	The right and left apex will be at the top of the screen with the atria below
	For IVC, angle the transducer medially or towards the patient’s right shoulder to view the right atrium. Slightly rotate the transducer counter clockwise. Tilt the transducer and soundwave beam slightly inferiorly or down to ‘open up’ or image the IVC in the long axis plane for measurements and IVC collapse/distensibility calculation. The measurement should be taken 1 to 2 cm in from the IVC/right atrium connection	The LV on the upper right side of the screen and the RV on the upper left side of the screen. If not sure which is the RV or LV, the tricuspid valve leaflets are always inserted closer to the apex than the mitral valve
		If the apex is leftward on the screen, angle or slide the transducer and soundwave beam medially
		If the apex is tilted rightward on the screen, angle or slide the transducer and soundwave beam laterally or rightward

IVC, inferior vena cava; LV, left ventricle; RV, right ventricle.

An incidental finding of ascites or a right pleural effusion may be visualized in this view. A left-sided pleural effusion, visualized in other views, is more frequently associated with a pericardial effusion.

### Parasternal long-axis view

The parasternal long-axis view (Table 2 and Figure 2) is used in the assessment of LV systolic function (normal, decreased, or severely decreased) [17]. This view is often technically easier to obtain due to patient positioning, reliable landmarks, and ease of maintaining a steady transducer position [1]. A small and hyperdynamic LV may indicate hypovolemia. A pericardial effusion may be appreciated. An ‘incidental’ finding of a left pleural effusion may be noted.

**Table 2:** **Parasternal views**

	**Parasternal long-axis view**	**Parasternal short-axis view**
Goal	LV size and function, RV size, RV wall motion, pericardial effusion, left pleural effusion. Mitral and aortic valve thickening. Significant valvular regurgitation - color Doppler	LV size, wall thickness, and function. RV size and function. Main pulmonary artery (dilated or not). Pericardial effusion. Aortic valve thickening. Significant valvular regurgitation - color Doppler
Patient position	Far left lateral, when possible	Far left lateral, when possible
Initial transducer placement	Second or third ICS, as close to the sternum as possible, with the transducer ‘marker’ directed at approximately 11 o’clock (towards right shoulder)	Second or third ICS, as close to the sternum as possible. From the PLAX view, rotate the transducer clockwise or to your right until the transducer ‘marker’ is at approximately 2 o’clock
Search for the best imaging ‘window’	Move the transducer up or down an ICS to search for the best view. Structures should appear horizontal on the screen. Image in the middle of the screen by adjusting transducer position	Mid-LV: angle the transducer slightly laterally and inferiorly or towards the patient’s left hip to image the mid-LV. Visualize the tips of the papillary muscles within the LV, below the level of the mitral valve. LV should appear round with RV clearly visualized. If the LV appears ‘egg-shaped’ or oblong and the RV is not visualized, slide the transducer ‘up’ an ICS
	If the apex appears to be positioned ‘uphill’ on the screen, slide the transducer ‘up’ an ICS	Main pulmonary artery: angle the transducer slightly laterally and slightly superiorly or toward the patient’s left shoulder with a slight clockwise rotation. You may need to move up an ICS, but in doing so may not be able to visualize the pulmonary artery bifurcation
	Increase the ‘depth’ to 20 to 24 cm to image the presence of a possible pleural effusion	
	Decrease the depth to approximately 14 to 16 cm, depending on the size of the heart, to fill the screen with cardiac structure	

ICS, intercostal space; LV, left ventricle; PLAX, parasternal long axis; RV, right ventricle.

### Parasternal short-axis view

The parasternal short-axis (PSAX) view (also known as the transverse view; Table 2) is used for the visual assessment of LV function at the mid-LV. The mid-LV (Figure 2) is identified when the tips of the papillary muscles are visualized as just ‘flickers’ of the mitral valve apparatus. The LV should appear round, with RV visualization at the mid-LV. The main pulmonary artery with the bifurcation may be visualized.

In the setting of hypovolemia or volume depletion, the LV may appear small and mitral valve leaflets may be visualized. The presence of regional wall motion abnormalities may be noted, though a comprehensive echocardiographic examination is indicated for evaluation of regional wall motion abnormalities. The RV may be evaluated for the presence of abnormal or paradoxical septal motion and the ‘D’ sign: flattening of the interventricular septum (RV pressure overload). This view is also used for presence of pericardial effusion. Incidental findings of clot/thrombus in the main pulmonary artery may be noted.

### Apical four-chamber view

Some authors caution about the use of A-4 views since off-axis views lead to erroneous interpretation of the LV and, particularly, RV size and function [1]. This view can be useful for identifying circumferential pericardial effusion. Assessment of RV size should be carefully made [38]. The RV/LV ratio may be used for evaluating RV size [48]: a RV/LV ratio <0.6 indicates normal RV size, a RV/LV ratio of 0.6 to 1.0 indicates moderate RV dilatation and an RV/LV ratio >1.0 indicates severe dilation [31,49,50]. The RV apex and LV apex must be at the top of the screen with the atria visualized directly below. An off-axis view may inaccurately cause the RV to appear dilated. This view is used for the assessment of pericardial effusion and for evidence of hemodynamic compromise. Circumferential pericardial effusion is often associated with a large pericardial effusion. Right atrial and/or ventricular diastolic collapse/buckling may be seen [2]. An incidental finding of a left pleural effusion may be visualized.

## Interpretation

Goal-directed echocardiography should always be interpreted in the context of clinically available hemodynamic and other clinically relevant information. Accordingly, a goal-directed echocardiography is not an anatomic investigation; it is a physiologic study or hemodynamic assessment (Table 3). Full interpretation involves linking goal-directed echocardiography findings to hemodynamic and other clinical information. We provide a practical ‘primer’ approach while full discussion of all aspects of hemodynamic instability, shock, and PEA arrest is beyond the scope of this review [51].

**Table 3:** **SHOCK: a practical mnemonic for the differential diagnosis of the spectrum of hemodynamic instability, shock, and pulseless electrical activity arrest**

**Type of shock**	**Goal-directed echocardiographic features**
Septic/distributive	Less severe shock - hyperdynamic. Severe shock - can be hypodynamic
Hypovolemic	Collapsing IVC, small ventricles, may be hyperdynamic
Obstructive	Tamponade: pericardial effusion, tamponade physiology
	Pulmonary embolism: large RV, septal shift/diastolic flattening
Cardiogenic	Decreased LV (±RV) function, dilated ventricles
K	Combinations (for example, septic plus hypovolemic, septic plus cardiogenic, and so on)
	Other kinds (adrenal insufficiency, neurogenic, and so on). This broader category is raised by the question ‘What does not fit?’

IVC, inferior vena cava; LV, left ventricle; RV, right ventricle.

Septic or distributive shock that is less severe or in the earlier stages may show a hyperdynamic LV and a smaller to normal size IVC with >50% collapsibility because septic shock is often initially associated with a degree of hypovolemia. More severe distributive shock may demonstrate impaired LV function [31].

Hypovolemic shock may demonstrate small ventricles, sometimes ‘near cavity obliteration’, due to a small and hyperdynamic LV, and a small IVC with wide respiratory variation which collapses >50% on inspiration in a spontaneously breathing patient [47,52,53]. If the patient is fully mechanically ventilated on positive pressure breathing, variation of the IVC diameter is associated with volemia [43,54]. Variation by more than 12% (the difference been maximal IVC diameter and minimal IVC diameter divided by maximal IVC diameter) suggests that cardiac output may increase significantly following volume infusion [55]. Whether fluid should be given, even when IVC diameter variation is large, is a clinical judgment that depends on other clinical variables [56]. Goal-directed echocardiography only supplements other clinical information. Patients who are responsive to volume loading can increase their cardiac output by ≥15% [31,54].

**Figure 3:**
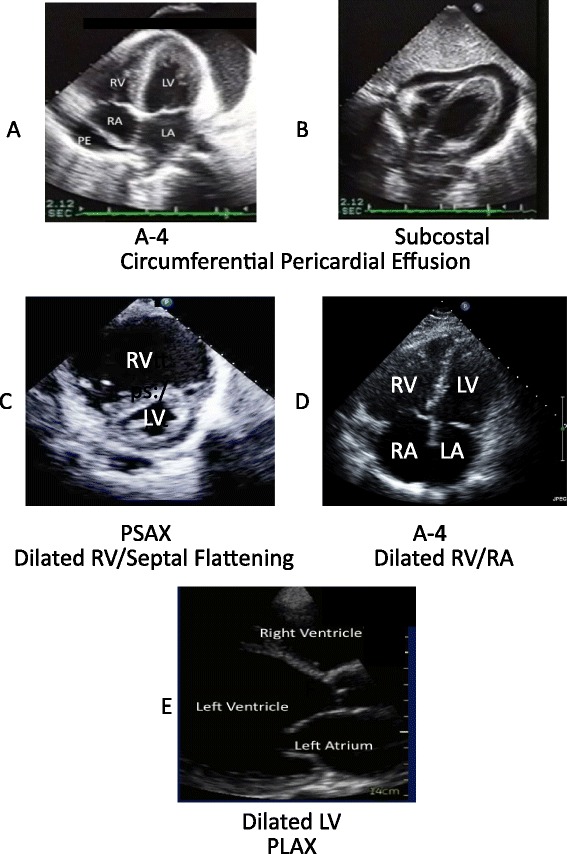
**Examples of pathological findings. (A)** Apical four chamber (A-4) view. Circumferential pericardial effusion. Left ventricle (LV), right ventricle (RV), left atrium (LA), right atrium (RA), and pericardium with a circumferential echocardiographic-free space or pericardial effusion (PE). **(B)** Subcostal view. LV, RV, LA, RA, and pericardium with a circumferential pericardial effusion. Diastolic right ventricular ‘buckling’ or collapse is demonstrated. **(C)** Parasternal short-axis view (PSAX) of the RV and the LV. The RV is dilated. The interventricular septum demonstrates ‘flattening’ or ‘displacement’, indicating evidence of RV pressure or volume overload. **(D)** A-4 view. Dilated RV, dilated RA, LV, and LA. **(E)** Parasternal long axis (PLAX) view of the RV, dilated LV, aorta, and LA.

Obstructive shock due to tamponade (evidence of hemodynamic compromise) may be observed in the A-4 view (Figure 3A) and the subcostal view (Figure 3B). These views will demonstrate a significant pericardial effusion (usually encompassing the heart) with right atrial wall or RV diastolic collapse. Delayed RV diastolic expansion may also be noted. These findings may indicate increased pericardial pressure [2]. The IVC is dilated indicating high right-sided filling pressures. ‘Swinging heart’ motion always indicates a large amount of pericardial effusion, but is not always associated with hemodynamic compromise. A patient may have tamponade physiology at times with a smaller amount of pericardial effusion. This is sometimes associated with faster pericardial fluid accumulation. It is beneficial to detect pericardial effusion in patients with PEA and near PEA arrest [32,57]. Pericardial tamponade is a clinical, not echocardiographic, diagnosis [2].

In the subcostal view, where you often image the largest amount of pericardial fluid accumulation, measure maximal echocardiographic-free space in diastole for potential pericardial ‘tap’ [58]. It is beneficial to note how many centimeters there are from the skin surface to the pericardium to help prevent inserting the pericardioscentesis needle too deep and close to the myocardium.

Obstructive shock due to acute pulmonary embolism will demonstrate a dilated RV (Figure 3C) and an RV/LV diameter ratio >1:1 with decreased RV contractile function [39,40,59]. An RV/LV ratio ≤0.6 is normal, an RV/LV ratio of 0.6 to 1.0 indicates moderate RV dilatation and an RV/LV ratio ≥1.0 (Figure 3D) indicates severe dilation [31]. Diastolic flattening of the septum may occur, leading to the ‘D’ sign or paradoxical septal motion, indicative of volume or pressure overload. The ‘D’ sign is seen in the PSAX view [38] (Figure 3C). There may also be systolic septal flattening [49]. The RV is sensitive to acute changes in afterload and may dilate under shock conditions [60]. A patient presenting with a pulmonary embolism may demonstrate an enlarged RV, a hypokinetic lateral wall, and a hyperdynamic apex (McConnell’s sign) [61,62].

The sensitivity of these findings to diagnose pulmonary embolism is 29% with goal-directed echocardiography and 51% with a comprehensive echocardiographic examination [2]. Thus, transthoracic echocardiographic examination is not sufficiently sensitive to detect or rule out pulmonary embolism. Goal-directed echocardiography is helpful to identify hemodynamic compromise in a patient having pulmonary embolism, which may be useful in directing the appropriate therapeutic intervention [39,63]. A patient may also have an increased RV/LV ratio in the setting of chronic and acute RV abnormalities such as chronic obstructive pulmonary disease (COPD), pulmonary hypertension, and RV myocardial infarction [2].

Cardiogenic shock will demonstrate a dilated LV (Figure 3E; consistent with underlying chronic heart disease) and/or significantly decreased LV function, possibly decreased RV function and a dilated IVC [5]. Additionally, marked valvular regurgitation can cause cardiogenic shock. Color Doppler imaging is useful for identifying significant valvular regurgitation but additional training is required for accurate use and interpretation [3,5]. LV function is graded as ‘not significantly decreased’ or ‘significantly decreased’, typically based on a visual estimate of the ejection fraction [64] (ejection fraction = (end-diastolic volume – end-systolic volume)/end-diastolic volume). These measurements and calculations must be performed by an experienced sonographer, cardiologist, or a physician with advanced echocardiographic imaging training and experience. With goal-directed echocardiography, LV function is graded as normal, decreased, or very decreased [37]. A minimal decrease in ejection fraction is not considered ‘significant’ since it is difficult to call unambiguously and is insufficient, alone, to cause cardiogenic shock.

Correct interpretation must incorporate the impact of preload and surrounding pressures from the lung and chest wall, the effect of afterload, and the effect of medications. For example, a ‘normal’ number for LV ejection fraction in a hypotensive patient treated with catecholamine infusions is not normal. Correct interpretation including these factors as well as decreased afterload and pharmacologically supported contractility may indicate substantially reduced innate LV contractility.

Goal-directed echocardiography can help distinguish between asytole, PEA arrest, and pseudo-PEA [33,34]. In pseudo-PEA there is echocardiographic evidence of ventricular contraction with no palpable pulses.

## RESP-F

Patients presenting with acute respiratory failure may share etiology and echocardiographic findings of SHOCK conditions. Hemodynamic instability may not yet be manifest in the patient who presents with acute respiratory failure, due, in part, to the increase in sympathetic tone that generally accompanies severe dyspneic states. There are substantial limitations using goal-directed echocardiography. In the absence of Doppler assessment, LV diastolic properties and filling pressures cannot be properly determined. This may require a more comprehensive echocardiographic examination.

Patients with acute exacerbation of an obstructive airways disease, such as COPD or asthma, may be hyperinflated so that most echocardiographic views are obstructed and often the heart is best visualized from a subcostal view. Features on goal-directed echocardiography that support the diagnosis of chronic COPD include evidence of RV hypertrophy and failure. Acute respiratory causes, such as asthma and pneumonia, may have few specific findings on echocardiographic examination. Pulmonary embolism leads to evidence of obstruction and right heart failure, as discussed above. Pericardial disease may be associated with ST changes and becomes important when it contributes to obstructive physiology. Acute ventricular dysfunction can result in chest discomfort, dyspnea, and pulmonary edema with hypoxemic respiratory failure and even ventilator failure. Myocardial infarction (ST changes) must be considered as a treatable and potentially very responsive cause. Ventricular dysfunction leading to heart failure is important in the differential diagnosis of acute respiratory failure and may be manifested in a goal-directed echocardiographic examination by the same findings that characterize cardiogenic shock.

## Conclusion

The role of goal-directed echocardiography in the critical care setting is continually evolving and becoming accepted as a beneficial modality in the treatment, care, and monitoring of the critically ill patient. It is time to work together as an interdisciplinary group to utilize this modality to the highest level of quality and accuracy for the benefit of our patients. Proper training is essential for the benefit and accurate diagnosis of patients. Poor training may encourage adverse consequences.

Diagnosis and treatment of critically ill patients will likely continue to improve with the use of goal-directed echocardiography with hand carried and portable ultrasound equipment.

## Abbreviations

A-4: Apical four chamber
COPD: Chronic obstructive pulmonary disease
IVC: Inferior vena cava
LV: Left ventricle
PEA: Pulseless electrical activity
PSAX: Parasternal short axis
RV: Right ventricle
